# ‘Real-life’ reduction in cholesterol with statins, 1993 to 2002

**DOI:** 10.1111/j.1365-2125.2007.03066.x

**Published:** 2008-02-20

**Authors:** Michael J Murphy, Li Wei, Alexander D Watson, Thomas M MacDonald

**Affiliations:** 1 Division of Pathology and Neuroscience Dundee, UK; 2 Medicines Monitoring Unit, Division of Medicine and Therapeutics, Ninewells Hospital and Medical School Dundee, UK; 3 Westgate Health Centre, Charleston Drive Dundee, UK

**Keywords:** coronary heart disease, effectiveness, lipid-lowering treatment, population cholesterol, statin prescribing

## Abstract

**AIMS:**

To evaluate the impact of lipid-lowering treatment on cholesterol concentrations in the setting of normal care.

**METHODS:**

This was a retrospective review of all cholesterol measurements made in Tayside, Scotland, between 1993 and 2002, linked to dispensed prescribing data for lipid-lowering drugs. It was conducted in the setting of normal care and included all patients who underwent cholesterol measurement. The main outcome measure was cholesterol concentration.

**RESULTS:**

A total of 401 489 cholesterol measurements were made on 128 240 patients over the study period. Measurements were categorized as treated and untreated according to whether patients were exposed to lipid-lowering treatment at the time the total cholesterol concentration was measured. Those categorized as untreated fell by 0.86 mmol l^−1^ (13.9%) and those categorized as treated by 1.45 mmol l^−1^ (23.5%). The difference between baseline and follow-up cholesterol concentrations in intention-to-treat patients was 1.53 mmol l^−1^ (24%) in 2002. In the same year, mean cholesterol concentration was 4.71 mmol l^−1^ (a fall of 1.65 mmol l^−1^ or 25.9%) in patients judged to be taking their lipid-lowering medication, compared with 5.20 mmol l^−1^ (a fall of 1.16 mmol l^−1^ or 18.2%) in those judged not to be taking treatment. Cholesterol fell by 0.38 mmol l^−1^ (6.3%) in a cohort of never treated patients (*n* = 33 679) between 1993 and 2002.

**CONCLUSIONS:**

The impact of lipid-lowering drugs on population cholesterol concentrations in the setting of normal care was significant and comparable with the cholesterol reductions seen in the setting of major statin trials, despite a significant proportion of the population receiving low dose treatment. In those subjects judged to be taking their medication, the benefits achieved were substantial. The impact of nondrug factors is indicated by the fall in population cholesterol seen in the absence of lipid-lowering treatment.

**WHAT IS ALREADY KNOWN ABOUT THIS SUBJECT**

- Statins reduce cholesterol concentrations and cardiovascular events in randomized clinical trials.
- Much less is known about their impact in the setting of normal care.

**WHAT THIS STUDY ADDS**

- This is the first study to assess the effectiveness of lipid-lowering treatment in the general population.
- We have also estimated the resultant impact on major vascular events.
- We have examined the actual and potential impact of lipid-lowering treatment.

## Introduction

Statin trials have ended the debate about whether lipid-lowering treatment can reduce cardiovascular events [1]. Much less is known about the effectiveness of lipid-lowering treatment in the setting of normal care. Although serial health surveys have documented rising use of lipid-lowering drugs and more aggressive lipid management [2, 3], population-based studies are required to establish the true impact of lipid-lowering drugs on cholesterol concentrations. We applied population-based, record-linkage methodology in order to assess the impact of lipid-lowering drugs on cholesterol concentrations in Tayside, Scotland, between 1993 and 2002.

## Methods

Our study was conducted using data from the Tayside Medicines Monitoring Unit (MEMO) database [4]. This population-based, record-linkage database contains several data sets including all dispensed community prescriptions, hospital discharge data, biochemistry results, and other data, all of which are linked by an unique patient identifier, the community health index (CHI). Data were anonymized, and methods approved by the Tayside Caldicott Guardians. The Tayside Committee on Research Medical Ethics also approved the study.

### Study sample

#### Total cholesterol measurements

All serum total cholesterol measurements between 1993 and 2002 were included in the study. Throughout the entire study period these measurements were subject to rigorous internal and external quality control procedures. Each measurement was categorized as treated or untreated according to whether patients were exposed to lipid-lowering treatment at the time of measurement (Table 1).

**Table 1:** Definition of measurements and patients

	Definition	Total numbers
**Treated measurements**	Patients were exposed to lipid-lowering treatment at the time the total cholesterol concentration was measured.	85 147
**Untreated measurements**	Patients were not exposed to lipid-lowering treatment at the time the total cholesterol concentration was measured.	316 342
**Intention to treat patients**	Patients who had at least two cholesterol measurements and were prescribed lipid-lowering drug treatment at some point after the first measurement.	20 179
**Never treated patients**	Patients had two or more cholesterol measurements in different calendar months, had an initial cholesterol concentration of greater than 5 mmol l^−1^ and were never treated with a lipid-lowering drug during the study period.	33 679

#### Lipid lowering treatment data

Data on lipid-lowering prescriptions in Tayside were available throughout the study period. Each dispensed lipid-lowering prescription had details of date of prescription, daily dose, amount and duration. We were thus able to identify whether patients were exposed to lipid-lowering treatment at the time the total cholesterol concentration was measured.

#### Intention-to-treat patients

Intention-to-treat patients were those who had at least two cholesterol measurements and who were prescribed lipid-lowering drug treatment at some point after the first measurement.

#### ‘Never treated’ patients

‘Never treated’ patients were defined as patients who had two or more cholesterol measurements in different calendar months, had an initial cholesterol concentration of greater than 5 mmol l^−1^ and were not treated with a lipid-lowering drug at any point during the study period.

#### Statistical analysis

All statistical analyses were carried out using SAS (version 8). Data were summarized as mean (SD) for continuous variables and number of subjects (%) for categorical variables. χ^2^ and *t*-tests and general linear model were performed to determine significant differences.

## Results

**Figure 1:**
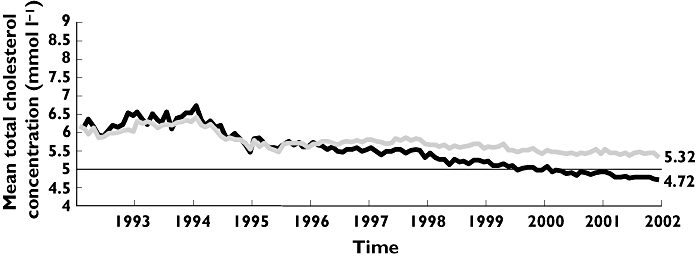
All cholesterol measurements in Tayside population, 1993–2002, categorized by exposure to lipid-lowering therapy at time of measurement (population not exposed to lipid-lowering therapy, (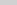); population exposed to lipid-lowering therapy, (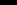))

Between 1993 and 2002, 401 489 cholesterol measurements were made on 128 240 patients. Of these, 15 064 were made in 1993 and 70 105 in 2002. Statin prescriptions increased from 5929 in 1993 to 118 488 in 2002, at which point they accounted for 98% of prescriptions for lipid-lowering drugs (*P* < 0.01) (Table 2). The proportion of total cholesterol measurements greater than 5 mmol l^−1^ was higher in women than in men (*P* < 0.01). Figure 1 shows trends in mean total cholesterol concentration between 1993 and 2002. Cholesterol fell by 0.86 mmol l^−1^ (13.9%) (*P* < 0.01) in the untreated group (6.18 mmol l^−1^ in 1993 (*n* = 1053) to 5.32 mmol l^−1^ in 2002 (*n* = 3533)), and by 1.45 mmol l^−1^ (23.5%) (*P* < 0.01) in the treated group (6.17 mmol l^−1^ in 1993 (*n* = 49) to 4.72 mmol l^−1^ in 2002 (*n* = 1422)). More aggressive treatment during the study period was shown by a rise in the percentage of patients on lipid-lowering drugs attaining a target cholesterol of less than 5 mmol l^−1^ (62.9% in 2002 compared with 34.6% in 1993), and by increased upward titration of statin doses over the same period (for example 24.7% people on simvastatin were on 20 mg or above in 2002 compared with 12.2% in 1993 (Table 2).

**Table 2:** Treated cholesterol measurements and doses of lipid-lowering drugs by year in Tayside population

	1993 *n* = 1045	1998 *n* = 8590	2002 *n* = 17 897
**Total cholesterol concentration (mean, SD)**			
** Total**	6.16 (1.49)	5.24 (1.11)	4.79 (1.02)
** Men**	5.95 (1.45)	5.31 (1.16)	4.63 (1.02)
** Women**	6.44 (1.48)	5.68 (1.14)	4.99 (0.99)
**Total cholesterol < 5 mmol l^−1^ (number, %)**			
** Total**	196 (18.76)	2976 (34.64)	11 258 (62.90)
** Men**	138 (24.04)	1889 (41.30)	6 625 (69.19)
** Women**	58 (12.31)	1087 (27.07)	4 633 (55.67)
**Statin doses (number, %)**			
** Simvastatin**			
** 10 mg**	475 (45.45)	2733 (31.82)	4 526 (25.29)
** 20 mg**	127 (12.15)	1281 (14.91)	4 429 (24.74)
** 40 mg**	0	231 (2.69)	1 282 (7.16)
** 80 mg**	0	6 (0.07)	110 (0.61)
** Pravastatin**			
** 10 mg**	70 (6.70)	294 (3.42)	381 (2.13)
** 20 mg**	55 (5.26)	194 (2.26)	674 (3.80)
** 40 mg**	0	65 (0.76)	661 (3.77)
** Atorvastatin**			
** 10 mg**	0	1160 (13.50)	2 107 (11.77)
** 20 mg**	0	629 (7.32)	1 589 (8.88)
** 40 mg**	0	274 (3.19)	985 (5.50)
** Fluvastatin**			
** 20 mg**	0	442 (5.15)	317 (1.77)
** 40 mg**	0	320 (3.72)	538 (3.01)
** Cerivastatin**			
** 100 μg**	0	196 (2.28)	0
** 200 μg**	0	86 (1.00)	0
** 300 μg**	0	29 (0.34)	0
** 400 μg**	0	5 (0.06)	0
**Total statin prescriptions (number, %)**	727 (69.57)	7945 (92.49)	17 599 (98.33)
**Other lipid-lowering drug prescriptions (number, %)**	318 (30.43)	645 (7.51)	298 (1.67)

### Intention-to-treat cohort and never treated cohort

**Figure 2:**
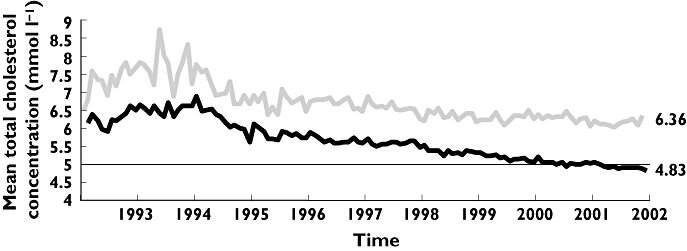
Baseline (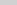) and follow-up (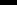) cholesterol concentration in subjects ever treated with lipid-lowering therapy (intention-to-treat patients), 1993–2002

**Figure 3:**
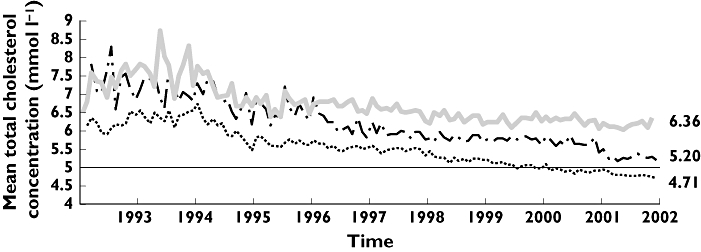
Baseline (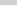) and follow-up cholesterol concentration in subjects ever treated with lipid-lowering therapy (intention-to-treat patients), 1993–2002, split by exposure status at the time of cholesterol measurement (follow up measurement not on lipid-lowering therapy, (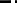); follow up measurement on lipid-lowering therapy, (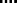))

In order more fully to estimate the impact of lipid-lowering treatment, we examined baseline and follow-up cholesterol concentrations in those untreated patients who were subsequently prescribed lipid-lowering treatment (intention-to-treat patients) (Table 1 and Figure 2). The mean follow up time was 4.6 years and the number of measurements and the mean value of total cholesterol in December of each year are shown in Table 3. Figure 3 shows that treated subjects (judged to be taking medication) had lower cholesterol than those untreated (judged not to be taking treatment). Finally, we observed a fall in cholesterol in a cohort of ‘never treated’ patients (Table 1). In this group cholesterol fell by 0.38 mmol l^−1^ (6.3%) (6.06 mmol l^−1^ minus 5.68 mmol l^−1^) between 1993 and 2002. Table 4 shows the total cholesterol changes (last measurement minus baseline measurement) for each patient during follow up. Greater reductions were seen in patients who were on lipid-lowering treatment at the time of the last measurement than in patients who were not on lipid-lowering treatment (*P* < 0.01). The mean concentration of total cholesterol changes between years 1 and 9 was 1.73 mmol l^−1^ to 1.83 mmol l^−1^ for patients who were on lipid-lowering treatment and 1.18 mmol l^−1^ to 1.38 mmol l^−1^ for patients who were not on lipid-lowering treatment.

**Table 3:** Baseline and follow up total cholesterol concentrations at December of each calendar year in the intention-to-treat patients

	Baseline total cholesterol concentration (mmol l^−1^)	Number of measurements	Follow-up total cholesterol concentration (mmol l^−1^)	Number of measurements
**1993**	6.82	130	6.50	50
**1994**	7.08	270	6.60	128
**1995**	6.28	309	5.61	187
**1996**	6.30	316	5.72	288
**1997**	6.41	637	5.59	620
**1998**	6.26	753	5.46	1242
**1999**	6.18	653	5.31	1219
**2000**	6.24	435	5.06	1482
**2001**	6.13	285	5.02	1456
**2002**	6.36	37	4.83	1830

**Table 4:** Total cholesterol changes during the follow up period in intention-to-treat patients

	Last measurement were not on treatment		Last measurement were on treatment	
Follow up time (year)	Number of patients	Mean change of total cholesterol concentration (mmol l^−1^) (SD)	Number of patients	Mean change of total cholesterol concentration (mmol l^−1^) (SD)
**1**	1643	−1.73 (1.22)	347	−1.18 (1.46)
**2**	1671	−1.84 (1.22)	522	−1.28 (1.54)
**3**	2051	−1.82 (1.30)	719	−1.30 (1.44)
**4**	2232	−1.72 (1.24)	772	−1.27 (1.57)
**5**	1857	−1.70 (1.37)	675	−1.18 (1.56)
**6**	1085	−1.80 (1.40)	404	−1.28 (1.33)
**7**	1011	−1.96 (1.38)	355	−1.40 (1.71)
**8**	1036	−2.06 (1.42)	340	−1.61 (1.71)
**9**	1648	−1.82 (1.38)	640	−1.38 (1.63)

## Discussion

This study is important for several reasons. First, it is the first to assess the effectiveness of lipid-lowering treatment on cholesterol concentrations in the general population since the publication of the first major statin trial in 1994. Second, because our data are population-based, the resultant impact on cardiovascular events can be estimated. Third, we have separately assessed the actual and potential impact of lipid-lowering treatment by examining whether or not patients were actually taking their drugs at the time of cholesterol measurement.

In patients who underwent cholesterol measurement in Tayside between 1993 and 2002, mean total cholesterol concentration fell steadily throughout the study period, and a widening gap emerged between treated and untreated groups. Various factors may have influenced prescribers and their ability to treat patients successfully to target, including the accumulating statin evidence base [5–9], the publication of evidence-based guidelines on management of cholesterol [10–12], and the advent of more potent statins during the period of study (atorvastatin was launched in the United Kingdom in 1997). Cholesterol also fell in the untreated group, in part because this cohort was continuously depleted as treatment thresholds fell, with subjects switching to the treated cohort who would previously have remained untreated. However, cholesterol also fell by 6.3% in a cohort of ‘never treated’ patients, suggesting that other factors, such as increased awareness of coronary heart disease prevention, with resultant changes in lifestyle, and cholesterol measurement in lower risk groups, may have contributed.

Cholesterol might reasonably be expected to be lower in treated patients compared with untreated, and this was clearly evident by the end of the study period, with cholesterol 0.6 mmol l^−1^ (5.32 mmol l^−1^ minus 4.72 mmol l^−1^) lower in treated patients in 2002. However, this substantially under-estimates the impact of lipid-lowering treatment. Throughout the entire period of the study, untreated patients who were subsequently prescribed lipid-lowering treatment had a higher mean total cholesterol concentration than the untreated group in general (for example 6.36 mmol l^−1^ compared with 5.32 mmol l^−1^ in 2002). Comparison of baseline and follow-up total cholesterol concentrations in treated patients shows a difference in mean total cholesterol of 1.53 mmol l^−1^ in 2002 (6.36 mmol l^−1^ minus 4.83 mmol l^−1^). Moreover, even this conceals the full potential impact of lipid-lowering treatment. We separated intention-to-treat patients into those who were or were not actually exposed to lipid-lowering treatment at the time of cholesterol measurement, and found, as expected that those who were actually taking treatment achieved lower cholesterol concentrations than those who were not (4.71 mmol l^−1^ compared with 5.20 mmol l^−1^ in 2002). This paper was not designed to address compliance issues, which have been examined in a separate study [13]. We understand that a dispensed community prescription does not mean that the patient is taking his treatment. However, this is the most accurate and widely used measure in observational studies. Thus in patients who were actually taking lipid-lowering treatment in 2002, mean total cholesterol was 1.65 mmol l^−1^ lower than in untreated patients (6.36 mmol l^−1^ minus 4.71 mmol l^−1^). Even if account is taken of other factors not directly related to lipid-lowering treatment which may have helped to reduce cholesterol, the remaining difference of 1.27 mmol l^−1^ (1.65 mmol l^−1^ minus 0.38 mmol l^−1^) is similar to the reduction in total cholesterol seen after 3 years in the Heart Protection Study (1.2 mmol l^−1^) [9].

Data from the Cholesterol Treatment Trialists' Collaborators allow us to estimate the relative risk reduction achieved in the Tayside population based on our findings. In that prospective meta-analysis, a 21% reduction in major vascular events was observed for every 1 mmol l^−1^ reduction in low density lipoprotein (LDL) cholesterol [1]. In the absence of triglyceride measurements we were unable always to calculate the LDL concentration for this study; this is one of the study limitations. However, we have previously shown in the same population [14] that total cholesterol and LDL are highly correlated. Also the Heart Protection Study [10] showed that there were similar percentage reductions with simvastatin between total cholesterol and LDL. Although our data are based on total cholesterol measurements, statins act predominantly to reduce LDL cholesterol and accounted for nearly all prescriptions for lipid-lowering drugs in 2002. However, statins also raise high density lipoprotein (HDL) cholesterol, and for this reason, our findings almost certainly under-estimate the relative risk reduction achieved. Based on our data, the 1.53 mmol l^−1^ reduction in total cholesterol seen in the intention-to-treat patients might translate into a 32% reduction in major vascular events. In those patients actually taking their lipid-lowering medication, the estimated risk reduction is even greater (35%), based on a 1.65 mmol l^−1^ reduction in total cholesterol. If patients with a 10-year risk of developing coronary heart disease (CHD) of at least 30% were targeted for lipid-lowering treatment (the minimum recommendation of the first Joint British Guidelines) [11], then the relative risk reductions we have estimated would translate into 10.5% fewer CHD events over 10 years in those taking their lipid-lowering medication.

In conclusion, the impact of lipid-lowering drugs (statins) on cholesterol concentrations in the setting of normal care is comparable with the cholesterol reductions seen in the major statin trials. In particular, in those subjects judged to be taking their medication, the benefits achieved are substantial. However, a role for other factors is indicated by the fall in cholesterol seen even in the absence of lipid-lowering treatment.

This work was supported by a grant from the Chief Scientist Office of the Scottish Executive (CZG/2/148), with pump-priming funding from EastRen (Project no. 106-04). The sponsors of the study had no role in study design, data analysis, data interpretation or writing of the report. All authors had full access to all of the data in the study.

Competing interests: M.J. Murphy has within the last 3 years received honorariums for lectures and advisory boards from Abbott, AstraZeneca, Fournier and Roche Pharmaceuticals. L. Wei declares no competing interests. A.D. Watson has within the last 3 years received honorariums from Novartis and Recordati. T.M. MacDonald has within the last 3 years received honorariums for lectures and advisory boards from Kaiser Permanente, Novartis, Pfizer, Recordati, Takeda and Speedel.
